# Urinary miR-16 transactivated by C/EBPβ reduces kidney function after ischemia/reperfusion–induced injury

**DOI:** 10.1038/srep27945

**Published:** 2016-06-14

**Authors:** Hsi-Hsien Chen, Yi-Fan Lan, Hsiao-Fen Li, Ching-Feng Cheng, Pei-Fang Lai, Wei-Hua Li, Heng Lin

**Affiliations:** 1 Division of Nephrology, Department of Internal Medicine, School of Medicine, College of Medicine, Taipei Medical University, Taipei, Taiwan; 2 Division of Nephrology, Department of Internal Medicine, Taipei Medical University Hospital, Taipei, Taiwan; 3 Department of Physiology, School of Medicine, College of Medicine, Taipei Medical University, Taipei, Taiwan; 4 Institute of Biomedical Sciences, Academia Sinica, Taipei, Taiwan; 5 Department of Pediatrics, Buddhist Tzu Chi General Hospital, Hualien, Taiwan; 6 Department of Emergency Medicine, Buddhist Tzu Chi General Hospital, Hualien, Taiwan; 7 Division of Pathology, Department of Internal Medicine, Shuang Ho Hospital, Taipei Medical University, New Taipei City, Taiwan; 8 College of Pharmacy, Taipei Medical University, Taipei, Taiwan

## Abstract

Ischemia-reperfusion (I/R) induced acute kidney injury (AKI) is regulated by transcriptional factors and microRNAs (miRs). However, modulation of miRs by transcriptional factors has not been characterized in AKI. Here, we found that urinary miR-16 was 100-fold higher in AKI patients. MiR-16 was detected earlier than creatinine in mouse after I/R. Using TargetScan, the 3′UTR of B-cell lymphoma 2 (BCL-2) was found complementary to miR-16 to decrease the fluorescent reporter activity. Overexpression of miR-16 in mice significantly attenuated renal function and increased TUNEL activity in epithelium tubule cells. The CCAAT enhancer binding protein beta (C/EBP-β) increased the expression of miR-16 after I/R injury. The ChIP and luciferase promoter assay indicated that about −1.0 kb to −0.5 kb upstream of miR-16 genome promoter region containing C/EBP-β binding motif transcriptionally regulated miR-16 expression. Meanwhile, the level of pri-miR-16 was higher in mice infected with lentivirus containing C/EBP-β compared with wild-type (WT) mice and overexpression of C/EBP-β in the kidney of WT mice reduced kidney function, increased kidney apoptosis, and elevated urinary miR-16 level. Our results indicated that miR-16 was transactivated by C/EBP-β resulting in aggravated I/R induced AKI and that urinary miR-16 may serve as a potential biomarker for AKI.

AKI is a multifactorial and multiphasic renal disease characterized by a rapid decline of renal function, resulting in renal tubular cell death that may occur through regulated apoptosis or necrosis. In 1992, Schumer *et al*. reported the first evidence of apoptosis in AKI^1^. Biochemically, renal I/R leads to the activation of caspases and regulation of apoptotic genes, including caspases and BCL-2 family proteins^2^,^3^. Necrosis is distinguished from apoptosis by the breakdown of the plasma membrane. The main events of necrosis involves stimulation of Toll-like receptors^4^,^5^, signaling through interferons^6^,^7^, and induction of a receptor-interacting protein–homotypic interacting motif (RHIM) domain to activate the kinase (RIPK3), phosphorylate mixed lineage kinase domain–like calcium dependent pathway to cause plasma membrane rupture^8^. In the kidney, necrosis was first suggested in renal ischemic AKI by showing a protective effect of necrostatin-1, an inhibitor of RIPK, on necrotic processing.

MiRs are small, non-coding RNAs that mediate mRNA cleavage, translational repression or mRNA destabilization^9^. There is growing evidence that miRs are dysregulated in urologic diseases, such as malignancies of the prostate, bladder and kidney^10^, exerting their effects as oncogenes or tumor suppressors^11^,^12^, and in renal diseases such as glomerulonephritis^13^ and AKI induced apoptosis^14^. Circulating miRs have been reported as biomarkers in critically ill patients with AKI. An array of circulating miRs can be reliably detected in the plasma of AKI patients and miR-210 had been reported as a strong and independent predictor of survival in critically ill AKI patients^15^. However, urine is one of the most easily accessible and noninvasive biofluids available in urology, nephrology, and primary care clinics but few reliable urinary miRs have been established as potential AKI biomarkers^14^. Our study attempted to find other urinary miRs that can be more stable than miR-494 which has been demonstrated to be a urinary biomarker for AKI^14^.

CCAAT/enhancer binding proteins (C/EBPs) are six-member family (α to ζ) of transcription factors. C/EBP-β is highly expressed in the various body tissues including kidney. Most studies have found that C/EBP-β is implicated in many biological activities including tumor progression, metabolic regulation, inflammation, endoplasmic reticulum (ER) stress and adipose differentiation^16^. No studies investigating the function of C/EBP-β in the kidney have been reported except for the ER stress induced renal C/EBP-β and the suppression of NF-κB–dependent gene expression in response to LPS in mice^17^. As for the relationship between miRs and C/EBPs, it has been reported that C/EBP-α regulated miR-34 and miR-30C in acute myeloid leukemia via NOTCH1 pathway^18^. However, similar studies showing any specific regulation of urinary miRs by C/EBPs in the kidney after I/R have not been reported. Here, we observed that C/EBP-β directly regulated miR-16 transcription in the kidney. I/R induced miR-16 blocked epithelium cells progression by inhibiting BCL-2 protein expression. Interestingly, urinary miR-16 was upregulated in patients with AKI. We also found that CEBP-β can elevate urinary miR-16 level in the kidney after I/R. Together, our study provides evidence that urinary miR-16 level is upregulated by the C/EBP-β-miR-16-BCL2 axis, which forms the molecular basis for AKI, and increased urinary miR-16 level may provide information in designing a strategy for the treatment of AKI.

## Materials and Methods

### Animal model for renal I/R

The C57BL/6 male mice, 8 to 10 weeks old, underwent bilateral renal artery occlusion for 45 minutes and reperfusion for the indicated time. Sham operation was identical to the treatment surgery except for pedicle clamping. All surgical procedures were approved by the Institutional Animal Care and Use Committee, Academia Sinica, Taipei, Taiwan. All experimental protocols were carried out in accordance with the approved guidelines. After renal I/R, mice were euthanized, urine were collected and the kidneys were homogenized for designated experiments.

### Patients and urine collection

A total of 18 human serum and urine samples were obtained immediately when patients were admitted to the Tzu Chi General Hospital (Hualien, Taiwan). These samples were collected from October 2013 to August 2014. This research project was approved by the Institutional Review Board of the Tzu Chi General Hospital and informed consents were obtained from all patients then samples were collected in accordance with the approved protocols and guidelines. The samples were collected from eleven critical patients who developed AKI (acute kidney injury), defined as ≧1.5 fold increase in serum creatinine in compliance with RIFLE-AKIN criteria. ICU control samples were collected from seven critical patients who did not develop AKI. The relevant information of these patients was listed in Table 1. In addition, serum and urine samples were obtained from six healthy volunteers. Urine samples of 25–35 ml were collected from the participating subjects. All samples were frozen at −80 °C until use.

### RNA isolation

Total tissue and cell RNA were extracted by Trizol (Invitrogen, NY, USA). Furthermore, the blood and urinary RNA of mice were extracted using the mirVana isolation kit (ABI, NY, USA), while the human urine RNA was extracted using the QiAMP circulating nucleic acid kit (Qiagen, CA, USA). The protocols for RNA isolation were conducted according to the manufacturer’s instructions.

### MiRs microarray profiling

The QiAMP circulating nucleic acid kit (Qiagen, CA, USA) was followed to isolate human urine total RNA, and subsequently analyzed with a Geniom Real Time Analyzer (GRTA) (Febit GmbH, Heidelberg, Germany) using the Geniom Biochip MPEA *Homo sapiens*. The total RNA quality was assayed on an Agilent Bioanalyzer 2100 (Agilent Technologies, Santa Clara, CA, USA). The quality control is done with the Agilent 2100 Bioanalyzer (Agilent Technologies, Santa Clara, CA, USA), using the RNA 6000 Nano Kit according to the manufacturer’s instructions. For each array, the RNA was suspended in Febit’s proprietary miRNA Hybridization Buffer (25 *μl* per array). Hybridization was done automatically for 16 h at 42 °C using the Geniom RT-Analyzer. The probes are designed as the reverse complements of all major mature miRNAs and the mature star (*) sequences as published in the Sanger miRBase v16.0 for homo sapiens. For each array, signal intensities were calculated using the Geniom Wizard Software (Febit GmbH).

### Quantitative qPCR and reverse transcriptase-PCR

Quantitation of the relative mRNA and miRNA abundance was performed using Applied Biosystems Step One Plus (Applied Biosystems, Foster City, USA). For the detection of miR-16 expression, we used TaqMan MicroRNA assays (Life Technologies) using small nuclear U6B (RNU6B) RNA as an internal standard. To quantify the amount of mRNA (BCL-2 and CEBP-β) and pri-miR-16, we used TaqMan Gene Expression Assay using *GAPDH* as an internal control. Samples were tested in triplicate, and differences of threshold cycles between target genes and house-keeping genes (GAPDH in mRNA and U6 in miRNA) were calculated by the 2^−ΔΔCT^ method using a control group as the calibrator according to the manufacturer’s user manual.

### Whole-mount *in situ* hybridization

Locked nucleic acid-modified miR-16 oligonucleotide probe (Exiqon, Vedbaek, Denmark) was labeled with digoxigenin. The IsHyb *In Situ* Hybridization kit (BioChain, CA, USA) was used according to the manufacturer’s protocol and nuclei were stained with Contrast green (KPL, MD USA) according to the manufacture’s protocol.

### Western blot analysis

Kidney and cell extracts were separated by SDS-PAGE and subjected to Western blot analysis using an ECL (enhanced chemiluminescence) kit (Pierce, Ill, USA). Antibodies used were anti-CEBPβ (1:500; Santa Cruz Biotechnology, CA, USA), anti-caspase-3 (1:500; Cell Signal Technology, MA, USA), anti-β-actin (1:10,000; Millipore, Darmstadt, Germany), anti-BCL2 and anti-lamin A (1:1000; GeneTex, CA, USA).

### Cytoplasmic and nuclear protein extraction

Cells and kidney tissues were processed for extraction of nuclear and cytoplasmic protein fractions according to the manufacturer’s protocols (Fermentas, MA, USA).

### Lentiviral production

All lentiviral vector stocks were generated by lipofectamine-mediated transfection of 293T cells (American Type Culture Collection, Manassas, VA, USA). The cells were cultured in Dulbecco’s Modified Eagle Medium (DMEM) (GIBCO, NY, USA) with 10% heat-inactivated fetal bovine serum (Omega, Tarzana, CA, USA). 293T Cells (4 × 106) were seeded into 10-cm^2^ culture dishes in 5.5 ml of the medium and transfected the following day with 2 μg of pMD-G plasmid, 8 μg of pCMV8.9 plasmid, and 12 μg of Lenti-miR16 vector plasmid (GeneCopoeia, MD, USA), antisense-miR-16 (SBI CA, USA) or Lenti-CEBβ. The medium was collected on day 2 and 3 post transfection and concentrated using the Vivapure LentiSELECT40 Kit (Sartorius Stedim Biotech, Aubagne Cedex, France).

### Intrarenal pelvic injection of lentivirus

The procedure was as described previously^14^. Mice were anesthetized with intraperitoneal pentobarbital (50 mg/kg). The renal artery, renal vein, and ureter were clamped at the same time just below the renal pelvis before transfection. Recombinant lentivirus, or PBS was slowly injected into the left renal artery with the use of a 30-G needle, subsequently the needle was removed and the ureter was declamped. After 2 weeks, mice were euthanized, and the kidneys were removed and homogenized for designated experiments.

### Histopathology

Mouse kidneys were fixed in 10% buffered formalin overnight at 4 °C and processed with paraffin fixation. Sections were stained with hematoxylin and eosin. Apoptosis in renal tissues was identified using the TUNEL (terminal deoxynucleotidyl transferase-mediated dUTP-biotin nick end labeling) assay with an ApopTag *In Situ* Apoptosis Detection kit (S7160, Chemicon, Darmstadt, Germany) and counterstained with DAPI (SouthernBiotech, AL, USA) following the manufacturer’s instructions.

### Measurement of biochemical parameters

At the end of reperfusion, 500-μl blood samples were collected via the tail vein. Samples were centrifuged at 6000 × *g* for 3 min to separate the serum from the cells. Biochemical parameters were measured in serum within 24 hrs.

### Assay for reporter activity

MiRNA plasmid was constructed using miExpress Precursor miRNA Expression clone (GeneCopoeia, MD, USA) containing murine precursor miR-16 DNA under the pEGFP plasmid. BCL2 3′UTR was constructed under the pRFP (red fluorescent protein) plasmid (from Chen, Chien-Chang, Academia Sinica, Taipei, Taiwan). Briefly, 293T cells (5 × 10^5^) were seeded into 6-well plates and transfected with pEGFP and pEGFP-miR-16 plasmids respectively. After 24 hrs, the cells were divided into four groups and each was transfected separately with the following four vectors including pRFP-BCL-2 3′UTR, scramble negative control, EGFP-miR-16 and miR-16 antisense. Finally, RFP/pEGFP fluorescent ratios were measured 48 hrs after transfection using a fluorescence microplate reader (Molecular Devices, CA, USA).

### *In vitro* hypoxia-reoxygenation experiment

Twenty-four hours after Lenti-pSin, Lenti-miR16, or Lenti-CEBPβ transfection, HEK293T cells were incubated under conditions of normoxia or hypoxia (1% O_2_) for 4 hrs and reperfusion for the indicated time. After transfection, cells were harvested for designated experiments.

### Luciferase assay

The putative promoters of the Homo sapiens miR-16 were predicted by miRstart (http://mirstart.mbc.nctu.edu.tw/) computational methods. 293T cells were transfected with pGL4, pGL4-miR16 promoter luciferase reporter vectors (pGL4-miR16-p0.5k, pGL4-miR16-p1k, pGL4-miR16-p2k) and Renilla vector using Lipofectamine 2000 (Invitrogen). After 24 hrs, the cells were harvested for firefly luciferase activity using Dual Luciferase Reporter Assay Kit (Promega). Each experiment was carried out at least in triplicate. The efficiency of transfection was normalized using the Renilla luciferase activity.

### Chromatin immunoprecipitation assay

One day after lenti-pSin or lenti-hCEBPβ transfection, 293T cells were incubated under conditions of normoxia or hypoxia (1% O_2_) for 4 hrs followed by 4 hrs of reoxygenation. The cells were fixed in 1% formaldehyde, and the ChIP assay was conducted using the Upstate protocol (Millipore). Chromatin was immunoprecipitated with anti-CEBPβ antibody (Santa Cruz Biotechnology). The purified DNA was detected using standard PCR with the following primer pairs for the *miR16* promoter region, 5′-GGTCCAACAGATAATTTACCCAACAAGGC-3′ (forward) and 5′-CCACCGCGTGGAGCCCTATAAAG-3′ (reverse).

### Statistical analysis

Values are expressed as means ± SEM from at least three experiments. The statistical significance was analyzed using ANOVA followed by the Tukey test for the *in vivo* experiments. Samples from the patients were analyzed using the Rank Sum test. A value of *P* < 0.05 was considered statistically significant.

## Results

### Increased urinary miR-16 concentration reflected AKI condition

**Figure 1:**
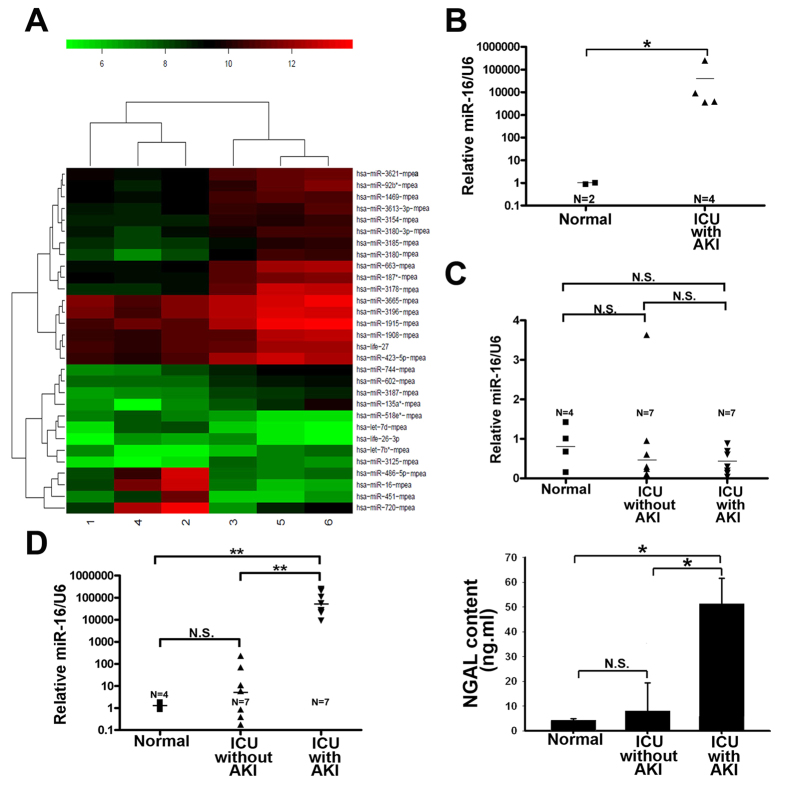
Novel set of acute kidney injury (AKI) related signature microRNAs (miRs) in the urine. (**A**) Heat map showing miRs significantly altered in the urine of AKI patients. MiRs expression signatures were obtained from microarray analysis of control and patients in intensive care unit (ICU) with AKI. Number 5 and 6 indicated normal, 1, 2, 3 and 4 indicated AKI patients in ICU. (**B**) Real time polymerase chain reaction (qPCR) analysis of urinary miR-16 in control and ICU patients with AKI. (**C**) Serum miR-16 levels were normalized to U6 RNA. Serum miR-16 values differed significantly between the control and ICU patients with AKI or between ICU patients with and without AKI. (**D**) Urinary miR-16 was measured by qPCR and neutrophil gelatinase-associated lipocalin (NGAL) were measured by ELISA kit (BioVendor, Candler, USA) in the control and ICU patients with or without AKI. *P < 0.05, N.S., no significant difference.

Previous study has shown that urinary miR-494 can be used as an indicator for AKI^14^. Therefore, we explored the possibility if new miRs could become alternative indicators for AKI. MiRs array profiling of urine samples from normal and AKI patients were compared. Figure 1A shows that 30 miRNAs were significantly modulated by more than two-fold, within these seven miRNAs were upregulated (let-7d, life-26-3p, miR-16, miR-451, miR-486-5p, miR-518e*, miR-720) and twenty one were downregulated. We were particularly interested in miR-16, a previously reported tumor suppressor in leukemia^19^, which showed the largest increase in AKI patients compared to normal subjects and also revealed consistent result with real time polymerase chain reaction (qPCR) (Fig. 1B). Furthermore, urine and serum samples were collected from the other seven patients in intensive care unit (ICU) with AKI, seven ICU patients without AKI, and four healthy volunteers. The serum miR-16 levels among the healthy volunteers, ICU patients with and without AKI did not differ significantly (Fig. 1C). However, the urinary level of miR-16 of ICU patients with AKI was significantly higher than those without AKI and normal volunteers (Fig. 1D, left). These results were consistent with the known urinary AKI marker NGAL (Fig. 1D, right). Thus, increased urinary miR-16 level reflects the condition of AKI.

### Urinary miR-16 was induced earlier than traditional kidney injury marker, urea or creatinine, in a mouse I/R renal failure model

**Figure 2:**
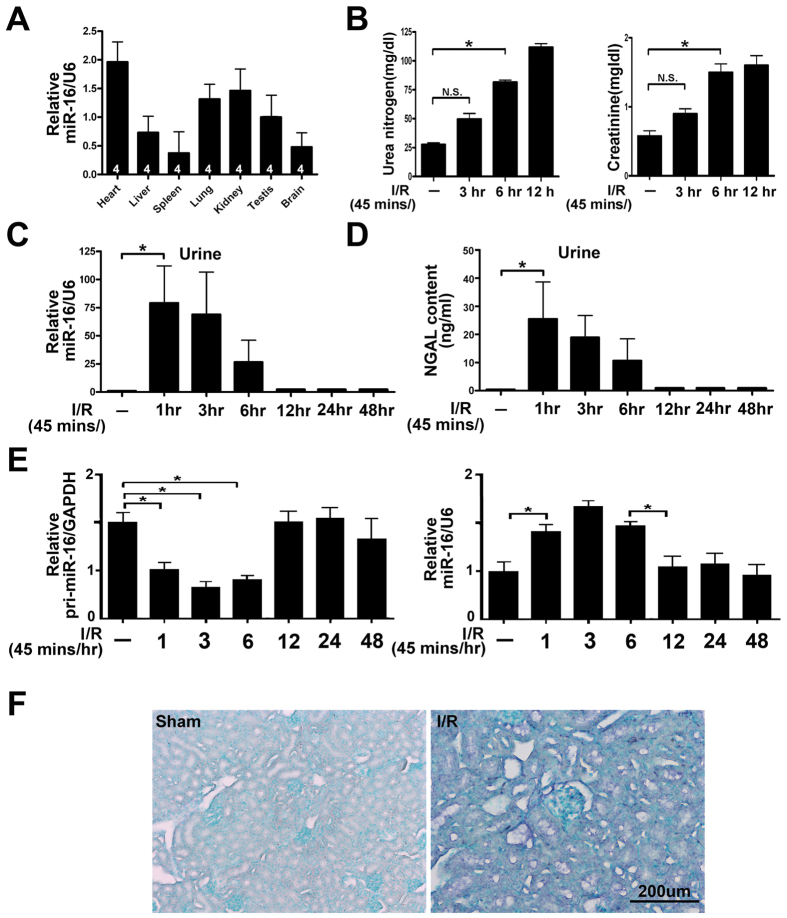
The expression of urinary microRNA(miR)-16 was induced by ischemia-reperfusion (I/R) injury in the kidneys. (**A**) The relative expression level of the mature miR-16 in various tissues of mice. MiR-16 levels were reverse transcribed utilizing miR-16 and U6 RNA-specific primers, and real time polymerase chain reaction (qPCR) was performed as described under “Methods”. Data are given as means ± SEM. n = 4 mice per group as shown in the diagram. (**B**) Time course of kidney functions assessed after I/R injury. *P < 0.05, n = 6 mice per group. (**C**) Time course of miR-16 level in the urine after I/R by qPCR assay. (**D**) Time course of urinary expression levels of neutrophil gelatinase-associated lipocalin (NGAL) in the urine measured by ELISA kit (BioVendor, Candler, USA) in the mice after I/R. *P < 0.05, N.S., no significant difference. (**E**) Time course of miR-16 or pri-miR-16 level in the kidney tissue after I/R by qPCR assay. (**F**) The localization of miR-16 in the mouse kidney after I/R (45 mins/3 hrs) injury was detected by *in situ* hybridization. Paraffin-fixed sections of mouse kidney were hybridized with the digoxigenin-labeled miR-16 probe and nuclei staining was stained by Contrast green. Figures are representative of three experiments performed on different days. Bar = 100, 200, 500 μm as indicated in the figures.

To delineate whether urinary miR-16 is related to the kidney, the content of miR-16 in various mouse organs was measured. Using semi-quantitative qPCR, we found that miR-16 was expressed highly in the heart, moderately in the kidneys, testes and lung, and lowest in the liver, brain and spleen (Fig. 2A). Our previous study indicated that the appearance of urinary miRs may result from kidney injury like I/R^14^, therefore, I/R mouse model was used. Figure 2B shows that serum urea and creatinine level were increased after ischemia followed by reperfusion for 3 hrs, however, urinary miR-16 was significantly increased after reperfusion for 1 hr. The elevated urinary miR-16 appeared earlier than the elevated serum urea and creatinine (Fig. 2C). These results were consistent with the known urinary AKI marker NGAL (Fig. 2D). Dicer and associated miRs have been reported to involve in I/R injury in the kidney^20^, and I/R injury is a major cause of AKI meaning miR-16 may come from immature miR-16, pri-miR-16, after I/R injury. Figure 2E (left) indicates that pri-miR-16 was rapidly declined from 1 hr lasting to 6 hrs after reperfusion, in contrast, the level of miR-16 was increased from 1 hr to 6 hrs after reperfusion in mice (Fig. 2E right). *In situ* hybridization also revealed that the expression of miR-16 was predominantly within the tubular epithelial cytosol after reperfusion for 3 hrs (Fig. 2F).

### The 3′UTR of BCL2 was a direct target of miR-16

**Figure 3:**
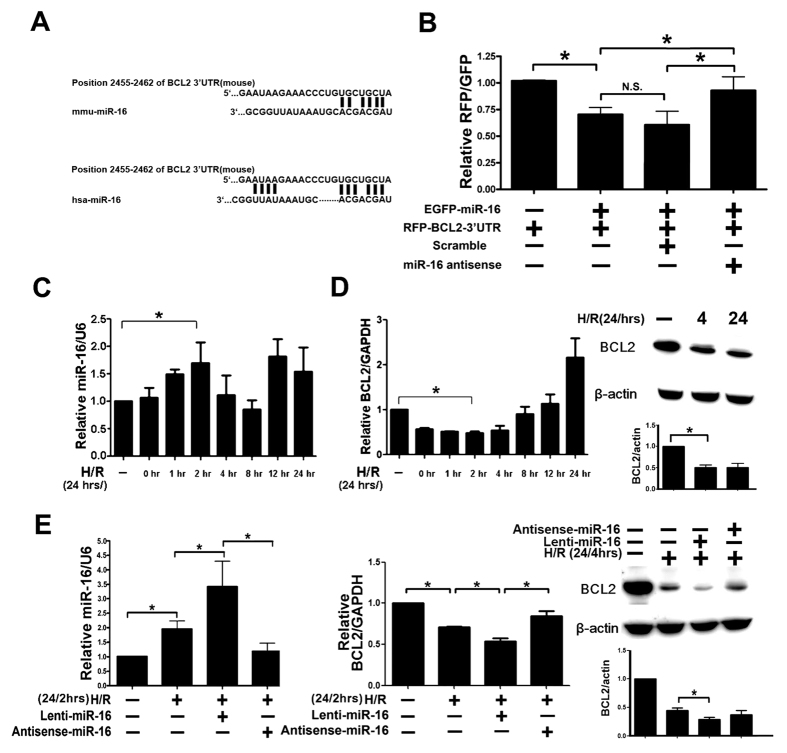
Overexpression of microRNA(miR)-16 inhibited B cell lymphoma 2 (BCL-2) expression *in vitro*. (**A**) Sequence alignment showing the relative position of the miR-16 binding site in the 3′UTR of BCL-2. (**B**) Expression of miR-16 decreased luciferase reporter gene activity in NRK-52 cells when linked to the targeted segment of the 3′UTR of BCL-2 and abolished miR-16 mediated decrease of luciferase activity after coinfected with antisense-miR16. (**C**) Time course of miRNA expression level of miR-16 in 293T cells after hypoxia/reoxygenization (H/R). (**D**) Time course of mRNA (reoxgenation period from 0 to 24 hrs) and protein level (reoxygenation period only 4 and 24 hrs) of BCL2 in 293T cells after hypoxia/reoxygenation (**E**) Real time polymerase chain reaction (qPCR) and protein expression levels detected marked induction of miR-16 which dramatically decreased BCL-2 levels in 293T cells after H/R (24 hrs/2 or 4 hrs) and abolished miR16-inhibited reaction by transfecting with antisense-miR16.

Apoptotic pathways are activated in the tubular epithelium cells by mitochondrial injury, ER stress, and I/R^21^. Using TargetScan (www.targetscan.org), we identified that nucleotides 2455–2462 of the 3′UTR of mouse BCL-2 and 2529–2536 of the 3′UTR of human BCL-2 are complementary to seed sequences of miR-16 (Fig. 3A). To study the direct interaction between miR-16 and BCL-2 transcription, the 3′UTR of BCL-2 downstream of the fluorescent reporter gene was cloned into the pRFP-C1 vector (RFP-BCL-2-3′UTR); precursors of miR-16 was constructed into pEGFP plasmids (pEGFP-premiR-16). NRK-52E cells were transiently co-transfected with both RFP-BCL-2-3′UTR and pEGFP-premiR-16, resulting in significant inhibition of fluorescent activity compared to RFP-BCL-2-3′UTR transfection alone. The fluorescent activity was reversed and returned to control level by miR-16 anti-sense transfection (Fig. 3B). The intrinsic relationship between BCL-2 and miR-16 in 293T cells after hypoxia/reoxygenation (H/R) also showed that the appearance of up-expression of miR-16, with maximum increase detected at 2 hrs post-reoxygenation, was accompanied by down-expression of BCL-2 after H/R. Although the expression of BCL-2 mRNA rebounded after 4 hrs of H/R (Fig. 3C,D left panel), the BCL-2 protein expression at 4 and 24 hrs post-reoxygenation was down-regulated (Fig. 3D right panel). Furthermore, both BCL-2 mRNA and protein expression at 2 and 4 hrs post-reoxygenation were further inhibited by miR-16 transfection and abolished by anti-sense miR-16 (Fig. 3E middle and right panel). These observations confirm that miR-16 binds to BCL-2 3′UTR and inhibits BCL-2 transcription and subsequent translation after H/R *in vitro*.

### Role of miR-16 in I/R-induced renal function and apoptotic response

**Figure 4:**
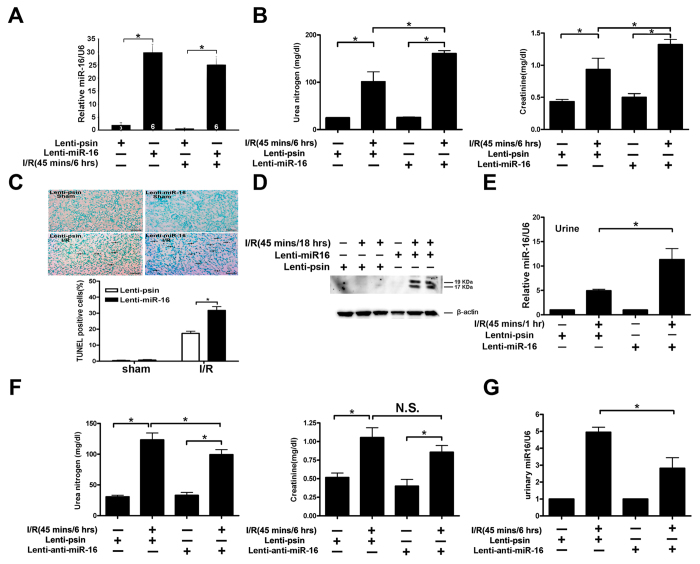
Gain or loss of microRNA(miR)-16 function attenuated or increased, respectively, kidney function in mice. (**A**) Quantitative analysis of miR-16 level after the kidney was infused with lenti-miR16 or lenti-pSin control (n = 6 mice per group as shown in the diagram). (**B**) Kidney functions assessed with or without miR-16 infusion following ischemia-reperfusion (I/R) injury. Bilateral renal arteries were clamped for 45 mins, and serum urea nitrogen and creatinine levels were measured 6 hours after reperfusion or sham surgery. Values are means ± SEM; n = 7 animals/group. *P < 0.05 compared to control groups. (**C**) Apoptotic kidney cells in mice infused with or without miR-16 using *in vivo* TUNEL staining. Without (left) or with infused miR-16 (right) mice underwent a sham operation (top) or 45 mins of renal clamping to induce ischemia, followed by 6 hours of reperfusion (bottom). TUNEL staining of representative kidney sections from each experimental group is shown. Colocalization of blue and brown staining in nuclei reflects apoptotic cells which are indicated with arrows. Bar = 50 μm. Proportions of TUNEL-positive renal epithelial nuclei to total nuclei in mice infused with or without miR-16 and subjected to the sham operation or I/R injury are shown. *P < 0.05; n = 7 animals/group. (**D**) Active caspase-3 protein expression in mouse kidney with or without lenti-miR-16 infection. Kidney lysates of mice subjected to the sham operation or I/R injury were probed with specific antibody against the cleaved, active form of caspase-3. Scanning densitometry was used for semi-quantitative analysis and compared to β-actin levels. Values are means ± SEM from three experiments. n = 3, **P < 0.01. (**E**) Urinary miR-16 level in mice after the kidney was infused with lenti-miR16 or lenti-pSin control following I/R. (**F**) Effect of Lenti-mediated gene transfer of antisense-miR-16 on I/R-induced renal function in mice. (**G**) Reduced urinal miR-16 content in mice infused with antisense-miR16 following I/R.

To further address the role of miR-16 in I/R injury, we compared the renal function (as assessed by blood urea nitrogen and creatinine) of mice with or without overexpression of either miR-16 or antisense-miR-16 in the presence or absence of renal I/R. As shown in Fig. 4A, overexpression of miR-16 significantly increased the mRNA level of miR-16 in the kidney without or with I/R compared to lentivirus GFP injection alone and overexpression of miR-16 also induced renal dysfunction (Fig. 4B), increased numbers of apoptotic TUNEL-positive tubular epithelial cells (Fig. 4C), elevated activity of the cleaved, active form of caspase-3 (Fig. 4D). High expression level of miR-16 was observed only in acute kidney injury mice (Fig. 2E,F). Thus, overexpression of miR-16 can induce kidney dysfunction which is accompanied by elevated miR-16 level in the urine. Figure 4E shows that significantly higher level of urinary miR-16 was detected in mice infused with lentivirus containing miR-16 compared to lenti-pSin controls after I/R. In contrast, overexpression of antisense-miR-16 in mice attenuated I/R-induced renal dysfunction compared to lenti-pSin controls (Fig. 4F). Likewise, urinary miR-16 level was reduced by *in vivo* lentivirus-mediated antisense-miR-16 gene transfer into the kidneys (Fig. 4G). These results suggest that miR-16 plays important pathophysiological roles in the kidneys after I/R and urinary miR-16 level may be a biomarker for kidney injury.

### C/EBP-β upregulated miR-16 transcription level

**Figure 5:**
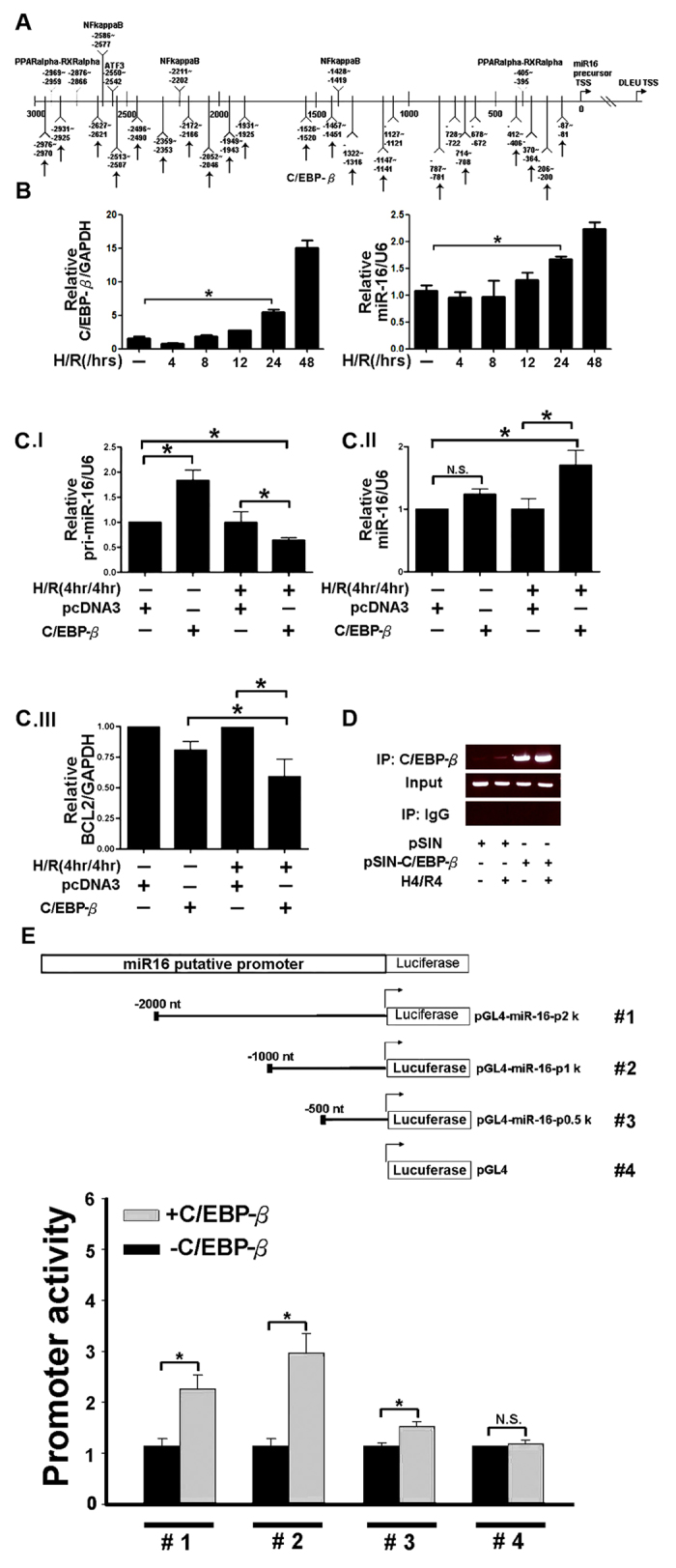
Gain of CCAAT enhancer binding protein beta (C/EBP-β) expression in renal epithelium cells upregulated the expression of microRNA(miR)-16 processing. (**A**) Alignment of the C/EBP-β binding site on the promoter region of the miR-16 genome. Vertical arrow indicated C/EBP-β binding site. (**B**) The mRNA expression level of C/EBP-β and miR-16 in 293T cells, after hypoxia/reoxygenization (H/R). (**C**) Increased expression of miR-16 accompanied with decreased B cell lymphoma 2 (BCL-2) levels in 293T cells transfected with C/EBP-β following H/R by real time polymerase chain reaction (qPCR). (**D**) Chromatin derived from K562-C/EBP-p42-ER cells was immunoprecipitated with anti-C/EBPβ and IgG antibodies. Recovered DNA was polymerase chain reaction amplified with primers specific for C/EBPβ-binding and the internal control. (**E**) Schematic description of the wild-type miR-16b conserved regulatory element construct containing C/EBP-β binding sites and construct with the deletion sequences, which were designated as CNS-Luc and mCNS-Luc, respectively. Luciferase activity of the wild-type (CNS-Luc) or different deletion (CNS-Luc) reporter gene in HEK 293T cells transfected with empty vector pcDNA3.1 or expression vector pcDNA3-LAP2. Data were analyzed using Student’s t-test (P < 0.05).

Several putative transcriptional factor binding sites including ATF3/CRE, PPARα-RXRα, NF-κB, C/EBP-β were found in the upstream promoter regions of miR-16 gene (Fig. 5A). To evaluate pri-miR16 level, 293T cells were transfected with ATF3, PPARα-RXRα, NF-κB, C/EBP-β plasmid separately, and only overexpression of NF-κB and C/EBP-β permitted evaluation of pri-miR-16 transcription level without H/R (Supplemental Fig. S1 and Fig. 5C I). To further confirm the relationship between C/EBP-β and miR-16 after H/R, both C/EBP-β and miR-16 were all increased in 293T cells after 24 hrs of reoxygention (Fig. 5B). After H/R, the pri-miR-16 level was dramatically reduced accompanied by an increase of miR-16 in 293T cells transfected with C/EBP-β compared to pcDNA3 control (Fig. 5C I,II). In addition, the target gene’s expression level of miR-16, BCL-2, was inhibited after H/R under C/EBP-β overexpression (Fig. 5C III). In addition, to examine whether the C/EBP-β protein was located in the nucleus and associated with the C/EBP-β binding region, a ChIP assay was performed. Figure 5D indicates that overexpression of C/EBP-β increased the amount of C/EBP-β protein recruited to the miR-16 promoter region compared to the scrambled control. To further determine the activity of C/EBP-β-induced pri-miR-16 transcription on the upstream of miR-16 genome promoter, serious deletion of miR-16 genome promoters were constructed (−2.0, −1.0 and −0.5 kb) and assayed for luciferase activity in 293T cells with or without C/EBP-β treatment. Figure 5E shows that significantly reduced luciferase activity was observed in the miR-16 promoter −1.0 to −0.5 kb region, but not in the −0.5 to −0.0 kb region (Fig. 5E). These results suggest that C/EBP-β interacts with the miR-16 regulatory region.

### MiR-16 was necessary for C/EBP-β to block BCL-2 and to induce kidney epithelium cell apoptosis resulting in kidney dysfunction

**Figure 6:**
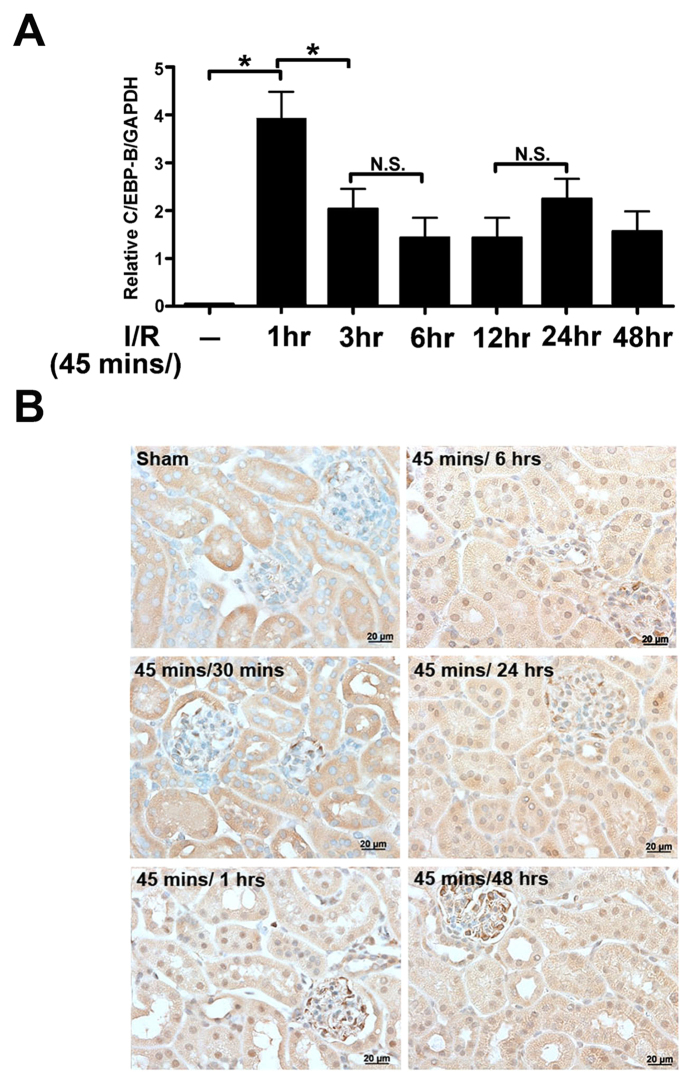
Renal CCAAT enhancer binding protein beta (C/EBP-β) induction upon ischemic-reperfusion (I/R) injury in wild-type (WT) mice. (**A**) Quantitative reverse transcriptase polymerase chain reaction analysis of C/EBP-β in kidney homogenates from I/R-treated WT mice. (**B**) Localization of C/EBP-β in the kidney tissue of sham-operated or I/R-treated WT mice by immunohistochemical staining. Arrows, C/EBPβ nuclear staining of tubular epithelial cells. Bar = 50 μm.

**Figure 7:**
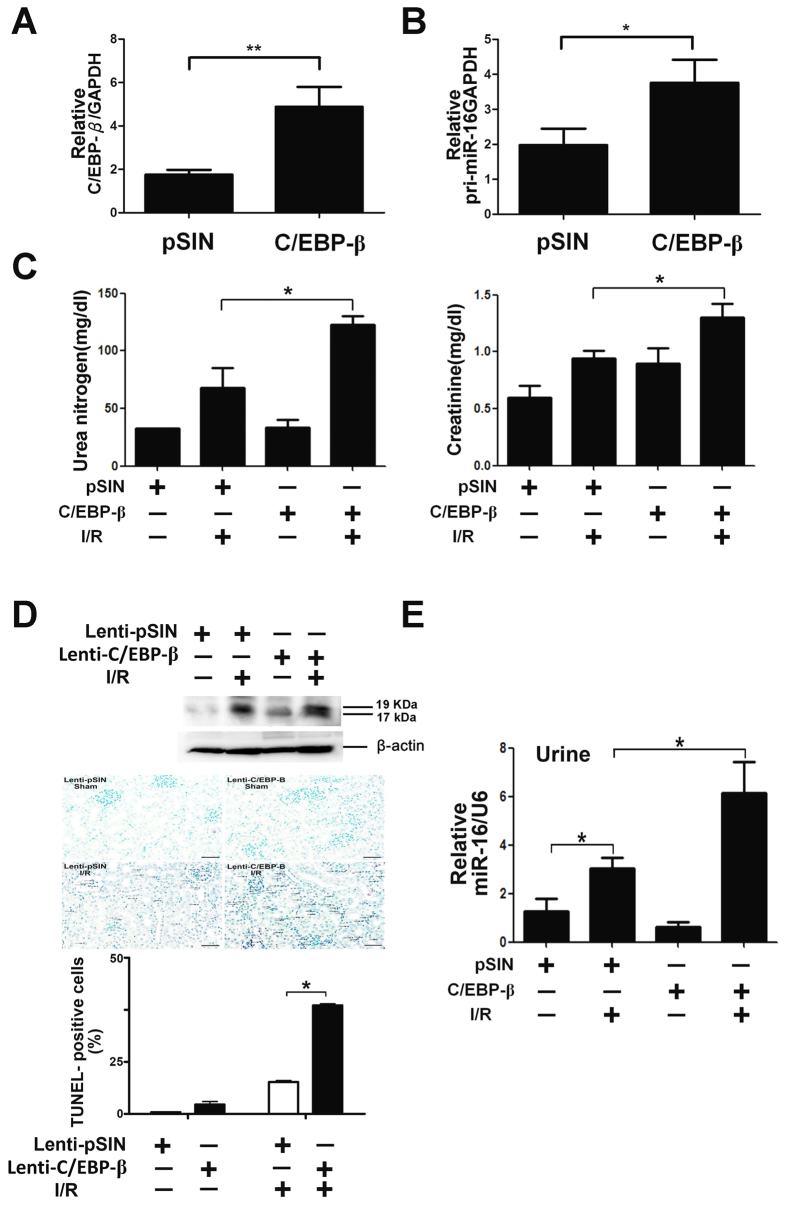
Effect of CCAAT enhancer binding protein beta (C/EBP-β) on ischemia-reperfusion (I/R)-induced renal dysfunction and apoptosis in mice. (**A,B**) Lentivirus-mediated of C/EBP-β and pri-miR-16 in the kidney. The recombinant lentivirus carrying C/EBPβ or GFP as controls (1 × 10^9^ viral particles per animal) was perfused into the wild-type kidney through renal artery. Two weeks after transduction, both C/EBP-β and pri-miR-16 mRNA level were assayed by real time polymerase chain reaction. (**C**) C/EBP-β is required for enhancing the I/R-induced renal dysfunction. Control (GFP or empty virus) or C/EBP-β mice underwent sham operation or I/R injury for 8 h. Blood urea nitrogen and creatinine levels, indicators for renal function, were measured. Data are means ± SEM (n = 5 in each group). *P < 0.05 and **P < 0.01, GFP versus C/EBP-β knockdown. (**D**) Apoptotic kidney cells in mice infused with or without C/EBP-β using *in vivo* TUNEL staining or kidney lysates were probed with specific antibody against the cleaved, active form of caspase-3. The full-length gels are presented in Supplementary Figure 7D. Colocalization of blue and brown staining in nuclei reflects apoptotic cells which are indicated with arrows. Bar = 50 μm. (**E**) Urinary miR-16 levels in mice with or without overexpression of C/EBP-β in the presence or absence of renal I/R.

Pri-miR-16 is transcriptionally regulated directly by C/EBP-β and targets BCL-2 by the *in vitro* assay. Therefore, whether C/EBP-β is expressed in the kidney during I/R, meaning that C/EBP-β blocks BCL-2 protein expression through upregulation of pri-miR-16 and that this is necessary for kidney dysfunction homeostasis, was investigated. qPCR indicated that C/EBP-β mRNA levels were significantly increased 1 hr after reperfusion and lasted for 48 hrs (Fig. 6A). Consistent with qPCR, immunohistochemistry revealed that increased translocation of C/EBP-β into nucleus was found 1 hr after reperfusion (showing purple staining) on both tubular epithelial and glomerular cells (Fig. 6B). Whether the pathophysiological role of C/EBP-β induced miR-16 in I/R injury is via inhibition of the BCL-2 pathway, the gain-of-function of lentivirus-mediated gene transfer was used to overexpress C/EBP-β in the kidneys of sham or I/R mice. As shown in Fig. 7A,B, both C/EBP-β and pri-miR-16 expression were increased in the kidney tissue infused with lentivirus containing C/EBP-β compared to the lenti-pSin (self-inactivating vector) indicating that miR-16 was indeed transcriptionally regulated by C/EBP-β. Furthermore, overexpression of C/EBP-β significantly increased I/R-induced renal dysfunction compared to without overexpression and the sham group (Fig. 7C). Enhanced renal dysfunction with overexpression of C/EBP-β was accompanied by increased numbers of apoptotic TUNEL-positive tubular epithelial cells, elevated activity of the cleaved, active form of caspase-3 and urinary miR-16 level (Fig. 7D,E). These results suggest that inhibition of anti-apoptotic gene BCL-2 by C/EBP-β induced miR-16 pathway together with increasing urinary miR-16 level may play important pathophysiological roles in the kidneys after I/R.

## Discussion

**Figure 8:**
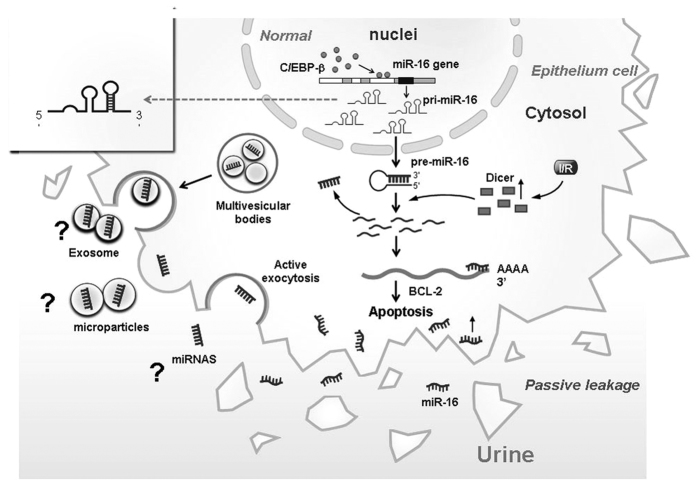
Schematic representation of regulation of kidney function by CCAAT enhancer binding protein beta (C/EBP-β)–microRNA-16(miR-16)-B cell lymphoma 2 (BCL-2) axis. During ischemia-reperfusion (I/R), C/EBP-β (top panel) transactivates miR-16 which, in turn, leads to BCL-2 repression and activation of epithelium cells apoptosis, resulting in kidney function loss. During CEBP-β alteration in kidney after I/R (bottom panel), low activity of CEBP-β fails to transactivate miR-16, which results in lack of BCL-2 inhibition and results in block of I/R induced kidney injury.

MiRs exert a prominent homeostatic plasticity in the kidney, such as tubular epithelium cells apoptosis, calcium handling^22^, podocyte function^23^ and renin-producing juxtaglomerular cells^24^, that are regulated by transcriptional factors^25^ but not including C/EBP family. Previous studies have shown that C/EBP-β is an important regulator in adipocyte differentiation, metabolism syndrome, ER stress and inflammatory cascade^26^, but studies of miRs regulation in the kidney have not been reported. Our results demonstrated a novel mechanism for C/EBP-β to regulate miR-16 in the kidney pathophysiology. Normally, C/EBP-β presents in the cytosol, and after I/R, it translocates into the nucleus and binds to the promoter region of pri-miR-16 genome resulting in the elevated level of pri-miR-16 in the nucleus and its subsequent translocation into the cytosol. After I/R, increasing amount of mature miR-16 are cleaved from pri-miR-16 by Dicer, which then inhibit one of the anti-apoptotic protein, BCL-2, leading to severe kidney injury (Fig. 8). We also showed that miR-16 was expressed at higher levels in the urines of AKI patients in the ICU compared to patients without AKI or normal individuals. In addition, urinary miR-16 is an earlier and non-invasive indicator compared to creatinine.

Most miR-16 studies have been focused on cancer and conflicting results have been reported. In breast cancer and esophageal squamous cell carcinoma, miR-16 suppressed cell apoptosis while promoted growth by regulating RECK, SOX6 or RPS6KB1^27^,^28^. But in gastric adenocarcinoma^29^ and human nasopharyngeal carcinoma^30^, miR-16 played an inhibitory role in cancer activation, suggesting that miR-16 targets different genes on various organs resulting in differential clinical outcomes. In our study on miR-16 targeted genes, we mainly focused on apoptosis signaling pathway gene, BCL-2. However, using integrative analysis of targetome (1,195 targets), TGF-β, PI3K-Akt, p53, GnRH, MAPK and ubiquitin mediated proteolysis signaling pathway were also found in the miR-16 regulatory network^31^. In addition, renal cell carcinoma related signaling pathways molecules, like PI3kinase, VEGFR2, VEGF, EIF4E and RAS, all are miR-16 target genes^32^. Because anti-apoptotic effects of PI3kinase, VEGFR2, VEGF, EIF4E, RAS may result from its anti-apoptosis or anti-oxidative effect after I/R, inhibiting these genes by miR-16 would result in greater apoptosis and hence larger damage to the kidneys. Therefore, these miR-16 related targeted genes may also involve in I/R induced nephropathy. Our *in vitro* H/R experiments revealed that elevated miR-16 level was accompanied by a decrease in BCL-2 protein expression (Fig. 3D right panel). However, BCL-2 mRNA expression rebounded at 24 hrs after reoxygenation (Fig. 3C,D left panel). We suggested that except for miR-16 there are many miRs like miR-15b, 302b, 497 may also will influence BCL-2 level *in vitro*^33^,^34^,whether BCL-2 be influenced by these miRs after H/R *in vitro* will be evaluated later. In addition, in Fig. 3E transfected with shRNA of miR-16 only *in vitro* did not effect on BCL-2 expression may also be effected by theses miRs except for miR-16. The other possibility suggested that up-regulation of Bcl-2 mRNA transcription but not link protein level after reoxygenation 24 hrs which may be regulated by some factors such as Cyclic AMP (cAMP) Response Element Binding Protein (CREB) and cAMP-Responsive CREB Coactivator-2^35^. But the results both from *in vitro* overexpression with lenti-miR-16 (Fig. 3E) and mice infused with shRNA-miR-16 (Fig. 4) definitely demonstrated the inhibition effect of miR-16 to BCL-2 and protected kidney function effect from I/R suggestion miR-16 actually plays an important pathophysiology role in kidney.

Many circulating miRs have been screened out for diagnosis and prognosis of various kinds of cancers including lung, colorectal, ovarian, pancreatic and other cancers^36^. It has been shown that miR-16-5p may be a prospective biomarker for gastric cancer and its progression by the variation of its plasma level^37^. However, most of these miR-16 studies failed to determine whether it is related to kidney dysfunction. In our study, results from clinical patients definitely indicated that up-expression of urinary miR-16 may be served as a biomarker for AKI patients. MiRs in the urine have been described as possible biomarkers for hepatotoxicity^38^, acute myocardial infarction^39^ or AKI^14^. In addition, Mall *et al*. showed that miR-16 and miR-21 are relatively stable in the urine under a variety of storage conditions, which supports their utility as urinary biomarkers^40^. We also demonstrated that miR-16 can be detected by using capped gold nanoslit SPR in a microfluidic chip after freeze-throw four times (Supplemental Fig. S2). Lorenzenz *et al*. also demonstrated that the levels of circulating miRs including miR-16, 24, 1244, 620, 320, 30d, let 7f and let 7b all were upregulated in patients with AKI compared to healthy controls or patients with acute myocardial infarction (non-AKI)^41^. High level of urinary miR-16 in AKI patients may come from the breakdown of tubular cells mediated by decreased BCL-2, resulting in apoptosis or necrosis^42^, which may explain why high level of urinary miR-16 were found in our clinical AKI patients (Fig. 1A,B). Whether other miRs, including 24, 1244, 620, 320, 30d, let 7f and let 7b, are all elevated in the urine of AKI patients require further study. The other possibility of high urinary miR-16 may result from increased miR-16 expression in the podocytes, which perturbs the actin cytoskeleton, and increases the release of exosomes containing miR-16 as previously reported^43^ (Fig. 8). Finally, Collino *et al*. showed that mesenchymal stromal cell-derived extracellular vesicles, also called exosome, carrying miRs or mRNA can repair AKI induced kidney injury^44^. Whether urinary miR-16 may exist as extracellular vesicle form called microparticle miR-16 (Fig. 8) and inhibit BCL-2 activity by endocytosis will be evaluated later.

Promoter analysis indicated that upstream genome region of miR-16 contains NFκ-β, PPARα-RXRα, ATF3/CRE and C/REBP-β binding sites, but except for NFκ-β and C/REBP-β, the other transcription factors failed to induce miR-16 (Supplemental Fig. S1). In the present study, we identified miR-16 as a novel direct target of C/EBP-β in tubular epithelium cells (Fig. 5). High expression of miR-16 regulated by NFκ-β in gastric cancer has been observed^45^, therefore, we cannot rule out the possibility that miR-16 regulated by NFκ-β may target BCL-2 during kidney I/R. Whether epigenetic modification of C/REBP-β is involved in miR-16 transcription, previous emerging evidence indicates that C/REBP-β can activate EF2-regulated gene by recruiting coactivator p300^46^ and acetylation by p300 resulting in C/EBP-β- mediated IL-6 and TGF-1 expression^47^. In this study, we demonstrated that miR-16 was regulated transcriptionally by C/EBP-β and whether other miRs are regulated by C/EBP-β resulting in kidney dysfunction will be evaluated by comparing the microRNA array analysis from the knock down of C/EBP-β in 293T cells after H/R 24 hours.

Our findings provide new insights for the C/EBPβ-mediated microRNA induced kidney dysfunction progression, which is the key step to disrupt renal epithelium cells resulting in elevated urinary miR-16 level. In this report, we show that C/EBP-β upregulates miR-16, and miR-16 blocks one of the anti-apoptotic genes, BCL-2, after I/R. We cannot rule out the possibility of miR-16 targeting other proapoptotic and antiapoptotic genes like programmed cell death 11 (PDCD11) and SOCS6. To our knowledge, this is the first report that shows the mechanism for urinary miR-16 levels enhancement by C/EBP-β after I/R in the kidney. Overexpression of epigenetic C/EBP-β by lentivirus can increase urinary miR-16 after I/R in the kidney. Finally, urinary miR-16 is stable and expressed earlier than creatinine, which makes it a useful indicator for AKI patients.

## Additional Information

**How to cite this article**: Chen, H.-H. *et al*. Urinary miR-16 transactivated by C/EBPβ reduces kidney function after ischemia/reperfusion–induced injury. *Sci. Rep*. **6**, 27945; doi: 10.1038/srep27945 (2016).

## Supplementary Material

Supplementary Information

## Figures and Tables

**Table 1:** Patient demographics for the study.

Patient Demographics	ICU without AKI	ICU with AKI
Patient characteristics	7	11
age (range)	20–89	30–87
gender	86% Men	75% Men
race	100% Asia	100% Asia
Comorbidities		
Hypertension (HTN)	2	1
Diabetes(DM)	1	1
Liver cirrhosis	0	1
Urinary tract infection	0	2
Pneumonia	0	1
Alcoholism	2	0
Shock	0	2
Gastroenteritis	0	1
others	2	5
AKIN stage (*N*)	I (0)	I (1)
	II (0)	II (5)
	III (0)	III (5)
APACHE II score (range)	17–24	13–49
Hospital clinic data		
creatinine (mg/dl)	0.8–5.9	1.5–21.3
BUN (mg/dl)	14–60	36–152
