# Supplementary material for: Urinary miR-16 transactivated by C/EBPβ reduces kidney function after ischemia/reperfusion–induced injury
Source: Sci Rep. 2016 Jun 14;6:27945. doi: 10.1038/srep27945 (PMC4906401; doi:10.1038/srep27945)

Urinary miR-16 transactivated by C/EBPβ reduces kidney function after ischemia/reperfusion–induced injury

Hsi-Hsien Chen, Yi-Fan Lan, Hsiao-Fen Li, Ching-Feng Cheng, Pei-Fang Lai, Wei-Hua Li, Heng Lin

Supplemental Figure S1. Potential transcriptional factors regulate pri-miR-16 transcriptional level. Quantitative RT-PCR analysis of pri-miR-16 level in 293 T cells after overexpression of NF-KB, PPARα and ATF3 respectively.

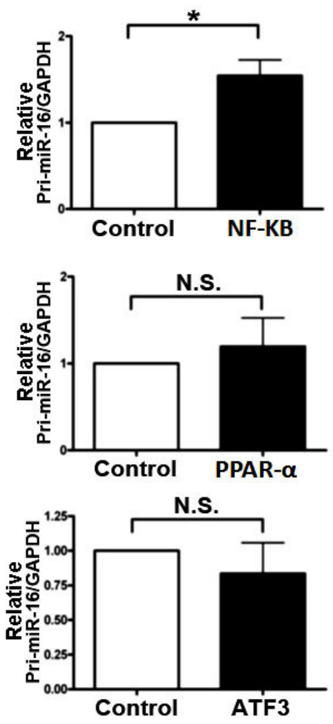

Supplemental Figure S2. Urinary miR-16 level stability assay. The double hybridization method to detect urinary miR-16 was applied to two AKI patients. The graph shows the fold-of-change of SPR response of two AKI patients (left Y axis).

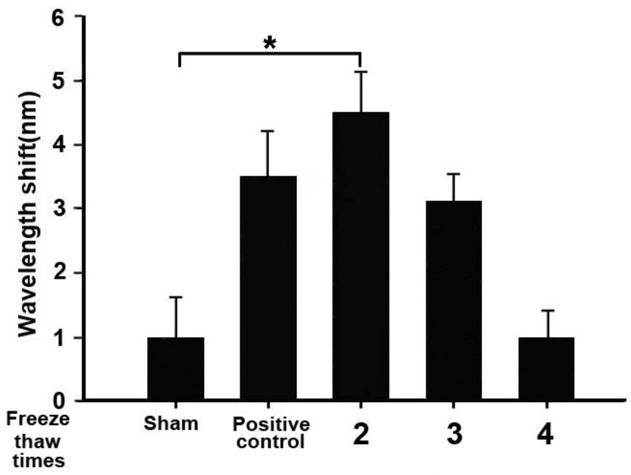

Supplemental Figure 7D. The full-length gels of Figure 7(D).

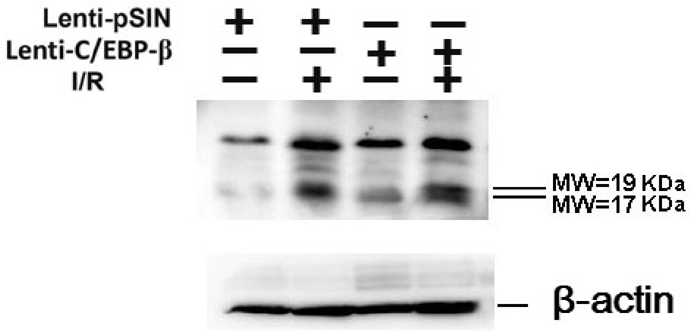

Supplement: Supplementary Information [file srep27945-s1.pdf]
